# Beyond Ebola Virus in the Largest Documented Bundibugyo Virus Outbreak

**DOI:** 10.3390/v18090925

**Published:** 2026-08-22

**Authors:** Damian Stodulski, Natalia Lopez-Escobar, Beatriz Cabanillas

**Affiliations:** 1 Department of Allergy, Instituto de Investigación Biosanitaria Hospital 12 de Octubre (imas12), Avenida de Córdoba s/n, 28041 Madrid, Spain; 2 Department of Biochemistry and Molecular Biology, Faculty of Chemical Sciences, Universidad Complutense de Madrid (UCM), 28040 Madrid, Spain

**Keywords:** Bundibugyo outbreak, Bundibugyo virus, Ebola virus, emerging infections, public health emergency

## Abstract

The ongoing Bundibugyo virus disease outbreak in the Democratic Republic of the Congo and Uganda has become the largest documented outbreak caused by this orthoebolavirus and already ranks as the second-largest Ebola disease outbreak recorded in history. This review highlights its major public health relevance, given its cross-border spread, substantial mortality burden, high clinical severity, infections among healthcare workers, and the lack of any approved vaccine or specific treatment for Bundibugyo virus disease. We discuss the epidemiological and virological significance of this exceptional event, the limitations of current diagnostic and therapeutic preparedness beyond Ebola virus, and the urgent need for broader filovirus-focused strategies, including improved diagnostics, genomic surveillance, candidate vaccines, antiviral platforms, and resilient local health systems. The outbreak illustrates how preparedness efforts centered mainly on the Ebola virus have left substantial gaps for other human-pathogenic orthoebolaviruses.

## 1. Introduction

The ongoing Bundibugyo virus disease outbreak in the Democratic Republic of the Congo (DRC) and Uganda represents the largest documented emergence of this orthoebolavirus and the second-largest Ebola disease outbreak recorded since 1976, a scale that underscores its exceptional public health impact and has rapidly made it a major regional and international concern. First reported to the World Health Organization (WHO) on 5 May 2026 following an outbreak of an unidentified highly fatal disease in the Ituri Province, the event was confirmed to be caused by the Bundibugyo virus and was declared by WHO a public health emergency of international concern (PHEIC) on 17 May 2026 because of its severity, cross-border spread, infections among healthcare workers, and potential for further international transmission. The absence of approved vaccines or specific treatments further exposes a critical weakness in global preparedness for less frequently encountered filoviruses [1,2,3]. WHO has assessed the risk in DRC as very high, the risk in Uganda and neighboring countries with substantial cross-border mobility as high, and the risk to the wider African region and globally as low [1,3].

Unlike the previous recognized Bundibugyo virus disease outbreaks in Uganda in 2007–2008 and in the DRC in 2012, the current event has unfolded on a scale that permits a broader reassessment of Bundibugyo virus disease within the landscape of Ebola disease preparedness [4,5]. Its magnitude, mortality burden, and occurrence in a setting where approved Ebola virus-focused vaccines and monoclonal antibodies cannot be directly extrapolated to Bundibugyo virus highlight the need to look beyond Ebola virus as the dominant model for filovirus preparedness [6,7,8]. This review therefore discusses the epidemiological and virological features of the current Bundibugyo virus disease outbreak, its historical context in relation to the two largest Ebola virus epidemics, clinical manifestations, and the current diagnostic, therapeutic, and vaccine-related gaps exposed by this emergency.

## 2. Epidemiological and Virological Features of the 2026 Outbreak

As of 15 August 2026, the outbreak had reached 4966 confirmed cases and 2327 deaths, corresponding to an overall reported case fatality ratio of approximately 47%. This total included 4945 confirmed cases and 2325 deaths in DRC, 20 confirmed cases and 2 deaths in Uganda, and one internationally exported case diagnosed in France after the patient returned from a humanitarian mission. At least 1059 patients had recovered, and the outbreak remained concentrated in Ituri Province, which accounted for more than 90% of confirmed infections [9]. The reported fatality ratio may nevertheless underestimate the true burden because deaths occurring before formal recognition of the outbreak remained under investigation [3]. The detection of an internationally exported case in France after returning from a humanitarian mission illustrates how contemporary mobility can rapidly internationalize the clinical consequences of a localized outbreak, even when sustained transmission remains concentrated in Central Africa [3,10].

**Figure 1:**
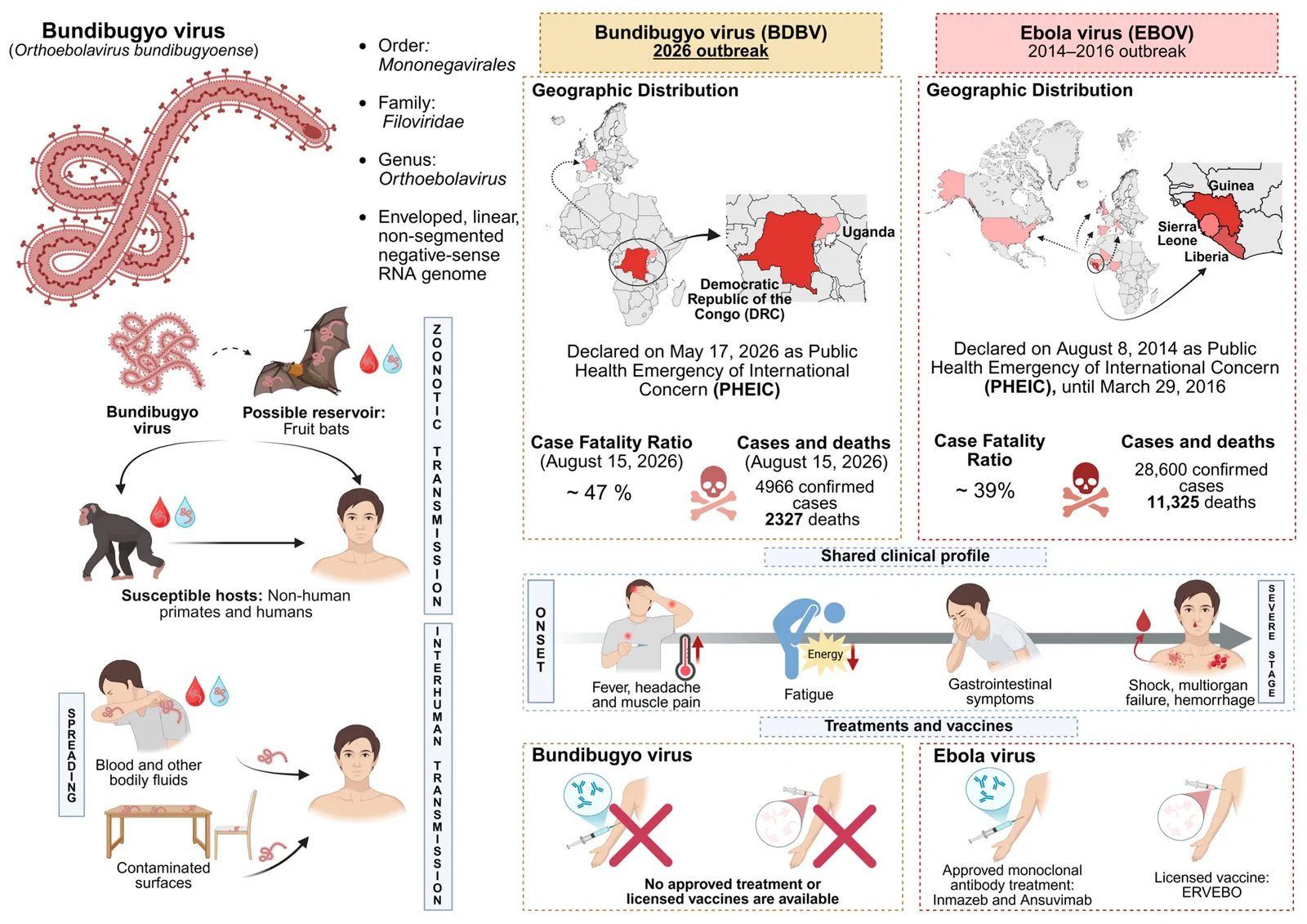
Schematic overview of Bundibugyo virus classification, zoonotic and human-to-human transmission, the 2026 Bundibugyo virus disease outbreak in comparison with the 2014–2016 Ebola virus disease outbreak, shared clinical features, and current therapeutic limitations.

Bundibugyo virus is an enveloped, non-segmented, negative-sense RNA virus classified within the order *Mononegavirales*, family *Filoviridae*, genus *Orthoebolavirus*, and species *Orthoebolavirus bundibugyoense* (Figure 1). The genus also comprises Ebola virus, Sudan virus, Taï Forest virus, Reston virus, and Bombali virus. Of these, the Ebola virus, Sudan virus, and Bundibugyo virus are the principal agents associated with substantial outbreaks of severe disease in humans [11,12].

Bundibugyo virus was first identified during the 2007–2008 hemorrhagic fever outbreak in western Uganda and subsequently caused a second recognized outbreak in DRC in 2012. The first event included approximately 131 reported cases, of which 56 were laboratory confirmed, whereas the 2012 outbreak involved approximately 38 confirmed infections [4,5]. Phylogenetic analyses placed viruses from the Ugandan and Congolese outbreaks in separate clusters, supporting independent zoonotic introductions rather than uninterrupted transmission between the two episodes. More detailed genomic reconstruction of the 2012 outbreak further suggested that its origin may have been more complex than a single spillover event, underlining the importance of sufficiently dense genomic sampling [4,13].

## 3. Historical Context, Transmission, and Clinical Features

The current event is the third recognized outbreak caused by the Bundibugyo virus and is already several times larger than the two previous outbreaks combined. It also ranks as the second-largest Ebola disease outbreak recorded since 1976, after the 2014–2016 West African epidemic and surpassing the 2018–2020 outbreak in the eastern DRC in both reported cases and absolute deaths, with both events caused by the Ebola virus. The West African epidemic (2014–2016) caused more than 28,000 reported cases and over 11,000 deaths mainly in Guinea, Liberia, and Sierra Leone (case fatality ratio: 39%), with imported cases in the United States, Spain, the United Kingdom, and Italy (Figure 1), whereas the eastern Congolese outbreak in 2018–2020 involved approximately 3470 cases and 2290 deaths with a case fatality ratio of 66% [7,8] (Table 1). These two outbreaks were declared PHEICs in 2014 and 2019, respectively; consequently, the current outbreak represents the third Ebola disease-related PHEIC and the first associated with the Bundibugyo virus [1,14] (Table 1).

In mortality terms, the current Bundibugyo virus disease outbreak has an overall reported case fatality ratio of approximately 47%, exceeding the crude ratio of around 39% reported during the 2014–2016 West African Ebola virus epidemic but remaining below the approximately 66% reported during the 2018–2020 eastern DRC Ebola virus disease outbreak [7,8] (Table 1). This value is also higher than the pooled historical case fatality ratio of 32.8% estimated for the Bundibugyo virus in a global meta-analysis, although still lower than the pooled estimate of 66.6% for Ebola virus [15]. Nevertheless, these comparisons should be interpreted cautiously, as mortality estimates vary according to case ascertainment, the inclusion of unresolved cases, delays between disease onset and clinical outcome, access to diagnosis and supportive care, and the availability of effective therapeutics. Thus, the current outbreak represents an exceptional mortality burden in absolute terms and a more severe reported mortality profile than previously expected for Bundibugyo virus disease.

Bundibugyo virus disease is considered a zoonotic infection, and fruit bats are suspected to constitute part of the natural reservoir, although the ecological source has not been definitively established [16,17]. Human infection is thought to begin after contact with infected wildlife or their tissues. Once introduced into humans, transmission can occur through direct exposure to blood, secretions, organs, or other bodily fluids from symptomatic or deceased individuals, or indirectly through contaminated objects, with unsafe burial practices further amplifying exposure. Healthcare settings are especially vulnerable when triage, isolation, personal protective equipment, environmental decontamination, or safe injection practices are inadequate [3,13].

The incubation period of Bundibugyo virus disease ranges from 2 to 21 days, and patients are not considered infectious before symptom onset. Initial manifestations, including fever, fatigue, myalgia, headache, and sore throat, are non-specific and overlap with malaria, typhoid fever, shigellosis, meningitis, bacterial sepsis, and other viral hemorrhagic fevers. Disease progression may involve vomiting, diarrhea, severe dehydration, electrolyte abnormalities, hepatic and renal dysfunction, shock, and multiorgan failure, while hemorrhagic manifestations occur only in a proportion of patients [3,18] (Figure 1). From an immunological perspective, severe filovirus disease is characterized not only by extensive viral replication but also by impaired innate antiviral defense, dysregulated cytokine and chemokine responses, and inadequate adaptive immune control [19,20].

Within this clinical and epidemiological context, the principal high-risk groups in the 2026 Bundibugyo virus disease outbreak appear to be healthcare workers, household caregivers, close contacts, and participants in funeral-related activities, broadly mirroring the exposure patterns observed during the 2014–2016 West African and 2018–2020 eastern DRC outbreaks caused by the Ebola virus [7,21,22]. In the West African outbreak, delayed recognition, weak health systems, limited access to protective equipment, and cultural burial practices increased risk among clinical staff, family caregivers, and communities with intensive contact networks. During the eastern DRC outbreak, these same groups remained vulnerable, while population mobility, armed conflict, and barriers to implementing control measures further increased exposure among border populations and residents of insecure areas [7,21] (Table 1).

Evidence from previous ebolavirus outbreaks suggests that women may experience higher exposure mainly through social and occupational roles rather than increased biological susceptibility. During the 2014–2016 West African Ebola virus disease outbreak, women were frequently involved in household caregiving and funeral preparation, all of which increased contact with infectious body fluids; care providers had an approximately three-fold higher risk of infection, 68% of care providers were female, and in one Sierra Leone transmission-chain analysis, 70.8% of cases generating secondary cases were women [23,24]. Similar gendered vulnerabilities were described during the 2018–2020 Ebola virus outbreak in eastern DRC, particularly in North Kivu and Ituri, where women’s roles in caregiving, ritual body preparation, health care, informal trade, and cross-border mobility shaped exposure patterns [25]. However, the role of women in transmission in the current 2026 Bundibugyo virus disease outbreak remains insufficiently documented, underscoring the need for systematic sex-disaggregated transmission data and gender-sensitive outbreak analysis.

## 4. Diagnosis, Clinical Management, and Investigational Countermeasures

Laboratory confirmation is indispensable because clinical assessment alone cannot reliably distinguish Bundibugyo virus disease from other endemic febrile illnesses. Molecular detection of viral RNA in blood samples remains central for confirming acute infection, and on 2 July 2026, WHO added the first molecular diagnostic test for Bundibugyo virus to its Emergency Use Listing, providing a quality-assured tool to support rapid case confirmation, timely clinical care, surveillance, and outbreak response [26]. This listing also marked an important step towards addressing diagnostic gaps exposed by the current outbreak. Nevertheless, the outbreak continues to underline the need for wider access to validated Bundibugyo virus diagnostics, including laboratory-based molecular assays, near-point-of-care molecular tests, and antigen rapid diagnostic tests, as well as sustained regional sequencing capacity. Clinical samples remain a major biological hazard and require rigorous biosafety procedures, validated inactivation, and appropriate transport conditions [3,5,27,28].

No licensed vaccine or specific treatment is currently approved for Bundibugyo virus disease. Early intensive supportive care therefore remains the foundation of clinical management and may substantially improve survival. Rehydration, correction of electrolyte and acid–base disturbances, hemodynamic monitoring, oxygen supplementation, treatment of concomitant infections, and management of renal, hepatic, and respiratory complications should be initiated as early as possible [3,18]. Clinical outcomes in Ebola disease are influenced not only by viral virulence but also by the timing and quality of supportive care and by the balance between effective antiviral immunity and harmful systemic inflammation [29,30].

WHO expert groups have recently identified several investigational countermeasures as priorities for clinical evaluation in Bundibugyo virus disease. Candidate treatments include the monoclonal antibodies MBP134 and Maftivimab, the antiviral remdesivir, and combinations of antibody-based therapy with remdesivir. The orally administered antiviral obeldesivir has also been proposed as a candidate for post-exposure prophylaxis among contacts, although its evaluation depends on timely and comprehensive contact tracing [31]. Vaccine candidates include a single-dose rVSV Bundibugyo vaccine and a ChAdOx1-based Bundibugyo vaccine [31,32,33].

By comparison, the landscape is more advanced for disease caused by the Ebola virus, for which a licensed vaccine is available. The 2014–2016 West African epidemic accelerated the development and clinical testing of Ebola virus-targeted vaccines [34], and during the 2018–2020 eastern DRC outbreak, rVSV-ZEBOV-GP was deployed at large scale through ring vaccination [33,35]. At present, ERVEBO, a single-dose rVSV-vectored vaccine for the prevention of disease caused by the Ebola virus, is the only licensed and WHO-qualified Ebola virus vaccine currently available [36,37]. The heterologous two-dose Ebola virus vaccine regimen Zabdeno/Mvabea was previously authorized in the European Union (EU); however, its EU marketing authorization was withdrawn on 1 May 2026 at the request of the marketing authorization holder for commercial reasons [33,34]. These advances do not eliminate the preparedness gap exposed by the 2026 Bundibugyo virus disease outbreak. Although recent immunogenicity studies suggest that Ebola virus vaccines may induce detectable cross-reactive antibodies against Bundibugyo virus [38], these responses appear lower than homologous EBOV responses, and evidence of clinical cross-protection remains limited and inconclusive [33,38] (Table 1).

Transmission control in the current 2026 Bundibugyo virus disease outbreak relies primarily on the classical non-pharmaceutical measures that shaped the response to previous ebolavirus epidemics, including rapid diagnosis, case isolation, contact tracing, safe and dignified burial, infection prevention and control in healthcare settings, and sustained community engagement. During the 2014–2016 West African Ebola virus disease outbreak, these measures were deployed at large scale in the absence of licensed vaccines during the early phase of the epidemic, and modeling studies showed that combined interventions, particularly case isolation, contact tracing with quarantine, and sanitary funeral practices, were required to reverse epidemic growth [39]. In the 2018–2020 eastern DRC outbreak, the same core package was complemented by vaccination and adapted response strategies in a setting complicated by insecurity, violence, community mistrust, and limited access to affected areas [35,40]. By contrast, the 2026 Bundibugyo virus disease outbreak exposes a major species-specific preparedness gap: although the principles of transmission control remain similar, licensed vaccines and effective monoclonal antibodies are not available. Consequently, containment of the current outbreak depends even more critically on early molecular confirmation, rapid isolation of symptomatic patients, strengthened healthcare infection prevention and control, safe burial practices, cross-border surveillance, and community trust until Bundibugyo-specific vaccines, post-exposure prophylaxis, or therapeutic countermeasures become available [6] (Table 1).

## 5. Conclusions

The 2026 Bundibugyo virus disease outbreak shows that filovirus preparedness cannot continue to rely predominantly on advances made for Ebola virus disease. Although previous Ebola virus epidemics reshaped the field of vaccines, therapeutics, diagnostics, and outbreak response, the current emergency demonstrates that this progress remains only partly transferable to other human-pathogenic orthoebolaviruses. As the largest documented Bundibugyo virus disease outbreak to date, it exposes a persistent gap between scientific preparedness for Ebola virus and the tools available for Bundibugyo virus.

The lesson from this outbreak is therefore not only that broader filovirus preparedness is needed but also that awareness must become more specific, anticipatory, and operational. Future efforts should avoid simply extrapolating from Ebola virus and instead prioritize Bundibugyo-inclusive diagnostic pathways, rapid in-country confirmation, regional sequencing capacity, and clinical research platforms that can be activated during outbreaks to evaluate candidate vaccines, antivirals, antibodies, and post-exposure strategies under real field conditions. Until validated Bundibugyo-specific countermeasures become available, control will continue to depend on early diagnosis, isolation, contact tracing, safe burial practices, and infection prevention and control.

This outbreak should therefore serve as a turning point towards broader filovirus preparedness, with greater emphasis on cross-reactive diagnostics, adaptable therapeutic platforms, and sustained genomic and clinical surveillance.

## Figures and Tables

**Table 1:** Comparative profile of the 2026 Bundibugyo virus disease outbreak and the two largest Ebola virus outbreaks.

Outbreak	Bundibugyo V irus 2026	Ebola V irus 2014 – 2016	Ebola V irus 2018 – 2020
**Causative** **virus**	*Orthoebolavirus bundibugyoense* (Bundibugyo virus)	*Orthoebolavirus zairense* (Ebola virus)	*Orthoebolavirus zairense* (Ebola virus)
**Geographic** **spread**	DRC with cross-border spread to Uganda and exported cases to Europe	West Africa (Guinea, Liberia, and Sierra Leone) with international spread to Europe and the US	Eastern DRC with export to Uganda
**Historical rank**	Largest documented Bundibugyo virus outbreak; second-largest Ebola disease outbreak overall	Largest Ebola disease outbreak recorded	Third-largest Ebola disease outbreak
**Approximate** **size**	4966 cases (up to 15 August 2026)	More than 28,000 cases	About 3470 cases
**Deaths/CFR**	2327 deaths; reported CFR about 47% (up to 15 August 2026)	Over 11,000 deaths; CFR about 39%	2290 deaths; CFR about 66%
**PHEIC status**	Declared a PHEIC in May 2026; first PHEIC caused by Bundibugyo virus	Declared a PHEIC in 2014; first PHEIC caused by Ebola virus	Declared a PHEIC in 2019; second and most recent caused by Ebola virus to date
**Main** **transmission** **setting**	Cross-border regional outbreak with infections among health-care workers and classic household/funeral exposure patterns	Rapid spread linked to weak health systems, delayed recognition, urban transmission, and burial practices	Similar transmission routes than 2014–2016 Ebola virus outbreak, but strongly complicated by armed conflict, insecurity, and mobile populations
**Therapeutics** **available**	No approved specific treatment; management relies mainly on supportive care, with investigational antibodies and antivirals under discussion	No licensed drugs during the outbreak; care focused on testing, barrier nursing, and complication management	Therapeutic agents were available and trialed, including monoclonal antibodies
**Vaccines** **available** **during** **response**	No licensed Bundibugyo-specific vaccine; candidates exist, but cross-protection from Ebola vaccines remains limited and inconclusive	No licensed vaccine early in the epidemic; vaccine development accelerated afterward	Response included field use of rVSV-ZEBOV ring vaccination and other vaccine strategies
**Community** **and response** **context**	Preparedness gap is species-specific: Ebola-focused tools do not transfer well to Bundibugyo	International response was initially delayed, and fragile health systems amplified spread	International response was faster and more tool-rich, but mistrust and limited local decision-making still hampered control

## Data Availability

All data generated or analyzed during this study are included in this published article.

## Abbreviations

The following abbreviations are used in this manuscript:

DRC	Democratic Republic of the Congo
WHO	World Health Organization
PHEIC	Public health emergency of international concern
MBP134	Mapp Biopharmaceutical 134
rVSV	Recombinant Vesicular Stomatitis Virus
ChAdOx1	Chimpanzee Adenovirus Oxford 1
